# Knowledge, attitudes, and practices regarding rabies in Grenada

**DOI:** 10.1371/journal.pntd.0007079

**Published:** 2019-01-29

**Authors:** Lindonne Glasgow, Andre Worme, Emmanuel Keku, Martin Forde

**Affiliations:** 1 Department of Public Health and Preventive Medicine, St. George’s University, St. George’s, Grenada; 2 Environmental Health Department, Ministry of Health, Social Security and International Business, St. George’s, Grenada; Wistar Institute, UNITED STATES

## Abstract

**Objective:**

While Grenada attained a zero-human-rabies case status since 1970, the authors conducted the first study to assess knowledge, attitudes, and practices that may contribute to this status as well as to receive feedback on the rabies control program in Grenada.

**Methodology:**

A cross-sectional survey was conducted in July, 2017 with 996 households on the mainland. A questionnaire was administered to collect information on knowledge of rabies and prevention, vaccination practices, perception of institutional responsibilities for rabies control, and evaluation of the anti-rabies program.

**Results:**

Of the 996 households, 617 (62%) had owners of animals that can be infected with rabies and were included in the analysis. Respondents were very aware of rabies as a disease that can infect animals and humans. The rate of participation in the vaccination program was 51.6% for pets and 38.0% for livestock. About 40% of respondents were knowledgeable about the extent of protection from the rabies vaccine. Respondents did not demonstrate exceptionally high levels of knowledge about animals that were likely to be infected with rabies, neither the anti-rabies programs that were conducted in Grenada. The three most frequent recommendations made to improve the rabies-control programs were: increase education programs, control the mongoose population, and expand the vaccination period each year.

**Conclusions:**

Conducting a comprehensive national rabies education program, expanding the vaccination program, and increasing the rate of animal vaccination are important steps that need to be taken to maintain the current zero-human-case status.

## Author summary

About 62% of households in Grenada owned animals that are susceptible to rabies. Pets, particularly dogs, were more commonly owned than livestock. Males were more likely to correctly identify animals that are susceptible to rabies. The level of participation in the animal vaccination program was low, especially among owners of livestock. A lack of knowledge about the free anti-rabies vaccination service and the length of time that it took to vaccinate animals were the most common problems associated with the low vaccination rate. Increasing education programs, controlling the mongoose population, and increasing the vaccination times per year were the most frequent recommendations to improve the Ministry of Health rabies control programs. The anti-rabies program remains a critical step to maintain the current zero-human-case status in Grenada. There is a need to also expand the vaccination program and increase the rate of animal vaccination.

## Introduction

Rabies is a zoonotic disease that is endemic in many countries, including in the Caribbean region [1]. The World Health Organization (WHO) reported that the disease kills tens of thousands of people every year [2]. Most deaths occur in developing countries and rural communities [3,4]. Global Alliance for Rabies Control (GARC) estimates approximately 55,000 people worldwide, mostly children, become infected and die annually [5]. Most human cases are as a result of bites from infected dogs. Bat, fox, and mongoose are also common hosts for rabies [6]. Notification of human cases of the disease declined in Latin America and the Caribbean from about 250 cases in 1990 to less than 10 cases in 2010 due to the implementation of dog rabies control programs [4]. Nonetheless, the high mortality rate among animal and human cases across the globe emphasizes the need for active surveillance and prevention programs [2].

Since 1983, the Pan American Health Organization (PAHO) has been providing technical support for countries to eliminate rabies [1]. In April, 2015, the first Regional Conference on Research and Surveillance on Emerging and Vector-Borne Animal Diseases in the Caribbean was held in Guadeloupe with a primary focus on rabies in the Caribbean [6]. Information was presented on the prevalence of rabies and prevention programs in the respective countries. The highest number of rabid animal cases per year was reported in Trinidad (6–10 cases) and Puerto Rico (>20 cases), while 1–5 cases per year were reported in Grenada, Belize, and Guyana, respectively [6]. Surveillance information was not provided for Suriname, Cuba, Haiti, and the Dominican Republic [6]. Review of the literature shows that few studies were published on knowledge, attitudes, and practices (KAP) relating to rabies in Caribbean countries. References were found for several studies in other regions, such as in African countries [7–9] and in the Americas [1,10–12].

In Grenada, the Ministry of Health (MOH) conducts several activities aimed at controlling the spread of rabies in animals and to maintain zero-human-case status. The programs conducted by the Ministry includes anti-rabies vaccination, stray dog control, mongoose trapping, public education, investigating reports of persons bitten by animals, and rabies surveillance. To date, no study was conducted to assess knowledge, attitudes, and practices that may contribute to maintain zero-human-case status or to assess risk factors for animal-human transmission of the disease in Grenada. The objective of this study, therefore, is to assess knowledge, attitudes, and practices in Grenada regarding rabies and to receive feedback from the public about the rabies control program in the MOH. The study was supported by PAHO, which is the sub-regional organization/body of WHO to provide support for countries in Latin America and the Caribbean to eliminate rabies. Based on the objective of the study, information was collected on the public’s response to vaccination programs, animal vaccination coverage, perceptions about institutional responsibilities for anti-rabies programs, evaluation of the Ministry’s anti-rabies programs, and knowledge about rabies and prevention. The findings can be used to guide the MOH in expanding and enhancing its anti-rabies programs to reduce the risk of the disease in animals and humans.

## Method

### Approval of the study

Ethical approval for the study was granted by the St. George’s University (SGU) Institutional Review Board (IRB).

### Study design and scope

The study design was a cross-sectional survey, administered to households in all parishes on the mainland in July, 2017. The State of Grenada includes the mainland, Grenada, and two smaller dependency islands, Carriacou and Petite Martinique. The mainland is divided into six parishes: St. Andrew, St. Patrick, St. Mark, St. John, St. George, and St. David. Rabies is endemic on the mainland, but not in Carriacou and Petite Martinique. Therefore, Carriacou and Petite Martinique were excluded from the study.

### Sample size

Using a hypothesized frequency of outcome at 50%, confidence limit of 5%, and the total of 33,670 households as determined by the Central Statistics Office (CSO) in Grenada, a minimum sample size of 570 was calculated. Given that a reference was not available of households with animals in Grenada, a total of 1000 households were decided for contact to ensure that at least 570 households were identified for inclusion in the study.

### Sampling

A multi-stage cluster sampling strategy was used with Enumeration District (ED) as the primary sampling units. In the first stage of sampling, the ED was randomly selected within the respective parish. Of the 283 EDs on the mainland, a total of 62 ED were randomly selected for inclusion in the study. In the second stage, households were randomly selected within the ED.

### Administration of the survey

All households in the ED on the mainland that were randomly selected for inclusion in the study were contacted. At the beginning of the survey, residents were asked whether anyone in the household owned pets and/or livestock (not including fishes, turtles, birds, snakes), which were considered as susceptible to rabies–that is, the animal can be infected with the rabies virus. The questions on KAP regarding rabies were only administered in households with owners of pets and livestock that were susceptible to rabies. The oldest person, above 18 years, who owned animals(s) in the household, was selected for the interview. If the owner was under 18 years, the head of the household, that was 18 years or older, was selected. One survey was administered per household in a face-to-face interview.

Written consent was required for participation in the survey. The survey was administered during the first two weeks in July, 2017. The questionnaire included primarily closed-ended questions and a few open-ended questions focusing on four main areas: participation in vaccination programs; perceptions about institutional responsibilities; evaluation of anti-rabies programs; and knowledge about rabies and prevention. A total of 46 questions were included and the responses were filled on the questionnaire by the interviewer. Each participant was assigned a numeric code.

### Analysis

Data were entered and analyzed using IBM SPSS software (V.24). Descriptive and inferential analyses were performed to determine:

- frequency of households with susceptible animals
- households with animals that were vaccinated, and use of vaccination services
- level of knowledge about rabies and prevention
- frequency of use of vaccination sites
- level of knowledge about the MOH’s rabies control program and satisfaction with the rabies prevention programs
- perception of benefits of vaccination and institutional responsibility for rabies control
- factors that restrict access to vaccination
- assessment of risk posed by animals with rabies to humans and other animals
- recommendations to improve the rabies control program and medium for education about rabies.

Chi square analysis was conducted to investigate relationships between demographic characteristics–education, gender, age group—of the respondents and KAP regarding rabies. Results that were statistically significant at alpha 0.05 are reported.

## Results

### Response rate and demographic characteristic of respondents

**Fig 1:**
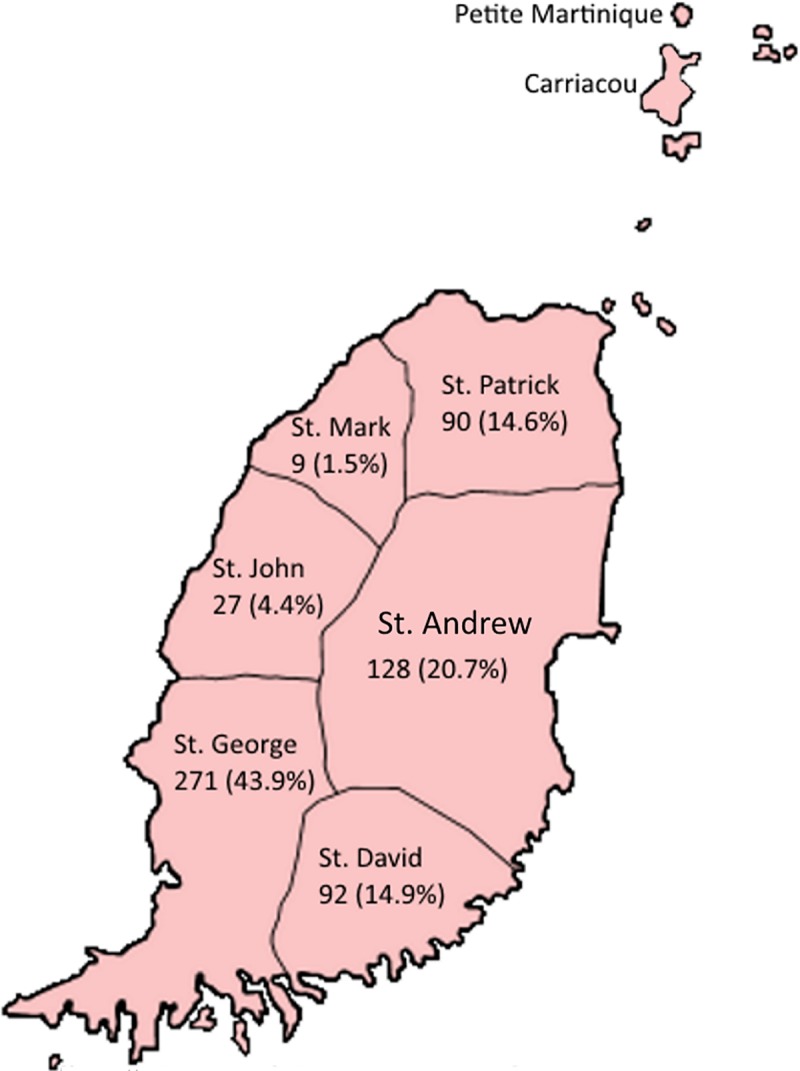
Distribution of respondents across the parishes.

Residents in a total of 996 households responded to the survey of which 617 (61.9%) households had owners of animals that were susceptible to rabies (animals that can be infected with the virus), distributed as follow: 271 (43.9%) in St. George, 128 (20.7%) in St. Andrew, 92 (14.9%) in St. David, 90 (14.6%) in St. Patrick, 27 (4.4%) in St. John, and 9 (1.5%) in St. Mark. Fig 1 shows the distribution of respondents across the parishes on the mainland in Grenada.

There were about equal proportion of males (307, 49.8%) and females (299, 48.5%) in the study. Representation was also fairly consistent across age groups except for a slightly higher percentage of respondents aged 46–55 years. Most of the respondents completed school at the primary (261, 42.3%) and secondary level (179, 29.0%). Table 1 shows the demographic profile of the respondents.

**Table 1:** Demographic characteristics of respondents.

Characteristic	Number	%	Characteristic	Number	%
Gender			Residence in the Parish		
Male	307	49.8	< 1 Year	30	4.9
Female	299	48.5	1–3 Year	30	4.9
Prefer not to say	11	1.8	>3 years	557	90.3
Total	617	100.0	Total	617	100.0
Age Group			Education		
18–25	98	15.9	Lower than primary school	19	3.1
26–35	107	17.3	Primary School	261	42.3
36–45	101	16.4	Secondary School	179	29.0
46–55	130	21.1	Vocational/Trade school	17	2.8
56–65	100	16.2	Community College	72	11.7
> = 66	81	13.1	University	55	8.9
Total	617	100.0	No response	14	2.3
Employment			Total	617	100.0
Unemployed	268	43.4	Occupation		
Employed full-time	189	30.6	Agriculture	60	17.8
Employed part-time	52	8.4	Professional	94	27.9
Self-employed fulltime	86	13.9	Trade/Business	46	13.6
Self-employed part-time	22	3.6	Skill Work	113	33.5
			Other	24	7.1
Total	617	100.0	Total	337	100.0

### Knowledge about animal susceptibility to rabies

The majority of households owned dogs, 323 (52.3%), while 86 (14.0%) of the households each had owners with cats, sheep and goats. Of the 617 respondents, 602 (97.6%) reported they had heard of rabies. School/work and electronic media (radio, television, internet/social media) were the primary sources of learning about rabies. In each case, more than 50% of respondents correctly identified dogs, mongooses, cats, and sheep/goats, as susceptible to rabies while 19 (3.1%) respondents stated they were not sure which of the animals were susceptible to the disease. Monkey, pig, bat, donkey, and cattle were correctly identified as animals that are susceptible to the disease by 30% or fewer respondents. Males were more likely to correctly identify sheep/goat χ^2^(2, N = 617) = 8.65, *p* = .013), pig χ^2^(2, N = 617) = 10.75, *p* < .01), monkey χ^2^(2, N = 617) = 18.22, *p* < .01), donkey/horse χ^2^(2, N = 617) = 10.24, *p* < .01), mongoose χ^2^(2, N = 617) = 22.93, *p* < .01), bat χ^2^(2, N = 617) = 13.94, *p* < .01), cattle χ^2^(2, N = 617) = 31.43, *p* < .01) as animals that were susceptible to the disease. Table 2 shows the number and percentage of respondents that correctly identified animals that are susceptible to rabies.

**Table 2:** Respondents that correctly identified animals susceptible to rabies.

Animal	Number (male)	%	Number (female)	%
Dog	286	46.4	262	42.5
Mongoose	252	40.8	188	30.5
Cat	206	33.4	184	29.8
Sheep/goat	205	33.2	163	26.4
Cattle	173	28.0	97	15.7
Pig	113	18.3	71	11.5
Monkey	104	16.9	54	8.8
Donkey/horse	94	15.2	56	9.1
Bat	77	12.5	38	6.2

### Knowledge of signs of rabies in animals

A few respondents, 23 (3.7%), stated they did not know how rabies was transmitted while 41 (6.6%) incorrectly stated the disease was transmitted through insect bites, 35 (5.7%) stated the disease can be transmitted through contact with an infected person, and 7 (1.1%) stated the disease can be transmitted through sneezing. The majority, 528 (85.6%), correctly stated that the disease can be transmitted by animal bite. Apart from identifying aggression as a sign of rabies in animals, less than 40% in each case correctly identified any of the other signs. Males were more likely to correctly identify aggression χ^2^(2, N = 617) = 13.27, *p =* .02), not afraid of people χ^2^(2, N = 617) = 12.01, *p* < .01), making unusual sounds/howling, bawling, bellowing χ^2^(2, N = 617) = 6.188, *p =* .05) the signs of rabies in animals. Table 3 shows the number and percentage of respondents that correctly identified signs of rabies in animals.

**Table 3:** Respondents that correctly identified signs of rabies in animals.

Signs	Number	%		
Aggression	232	37.6	186	30.1
Salivating/dribbling	130	21.1	105	17.0
Not afraid of people	85	13.8	46	7.5
Gaping	48	7.8	43	7.0
Making unusual sounds/howling, bawling, bellowing	45	7.3	24	3.9
Not eating/drinking	9	1.5	8	1.3

### Knowledge and attitude regarding animal vaccination

About two-third of respondents, 462 (74.9%), correctly identified vaccination to prevent transmission of rabies. A small percentage of respondents, 15 (2.4%), felt that rabies was not preventable, and 72 (12%) did not know of any way to prevent the disease. Respondents also demonstrated little knowledge about the protection that was provided for animals from vaccination. A total of 368 (62.2%) respondents stated they did not know how for long the vaccine protected the animals. Only 79 (13.3%) respondents correctly stated 1 year.

### Vaccination practices

**Fig 2:**
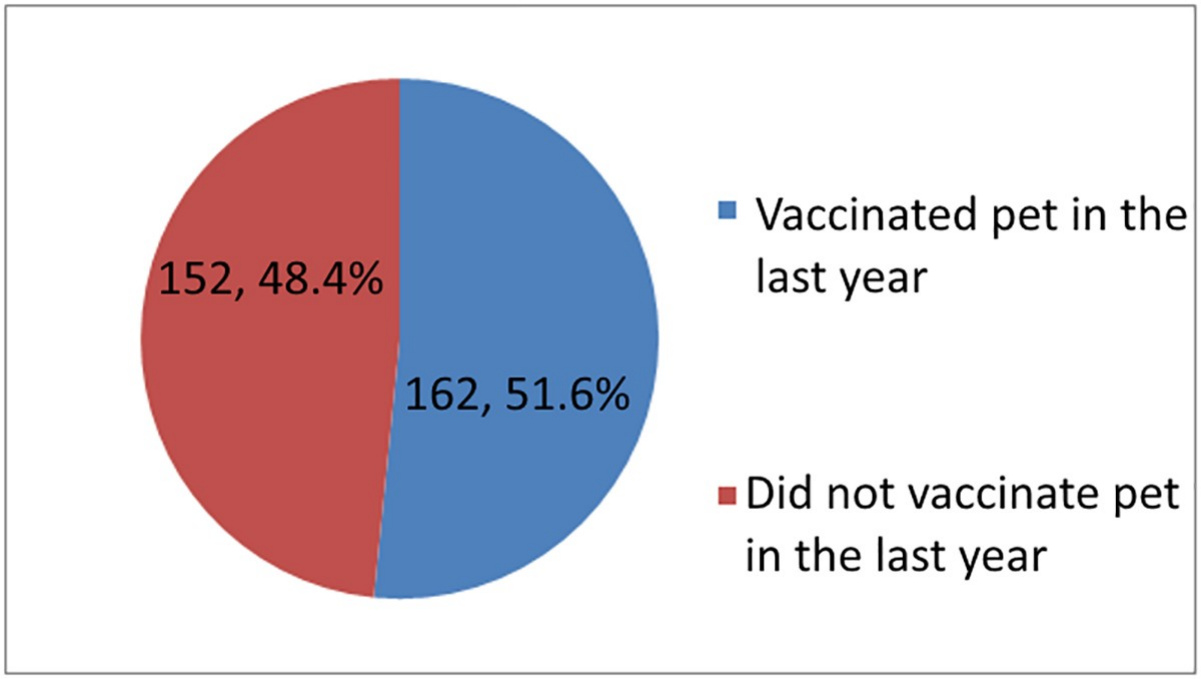
Number of respondents that vaccinated pets in the last year.

**Fig 3:**
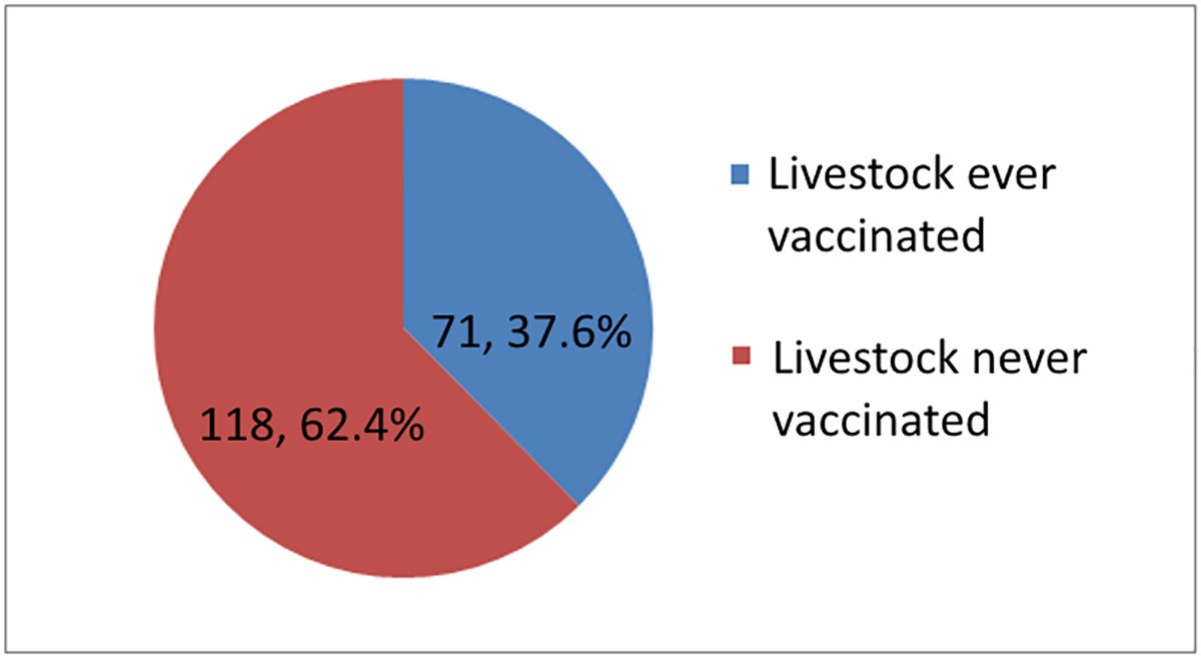
Number of respondents that vaccinated livestock at any time.

**Fig 4:**
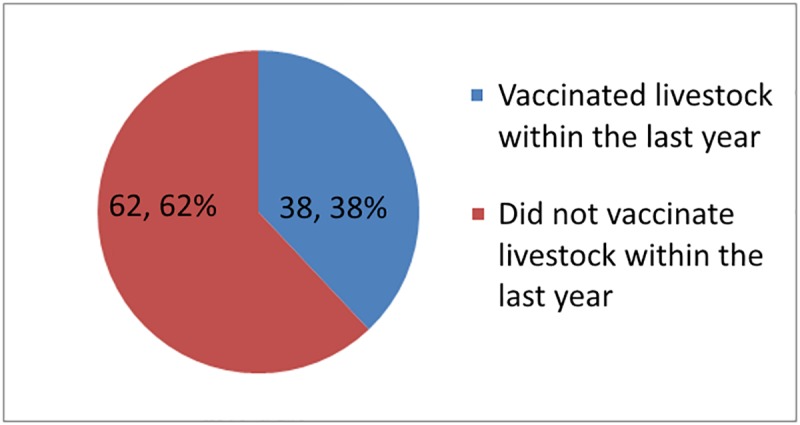
Number of respondents that vaccinated livestock in the last year.

Fig 2 shows (162) 51.6% of respondents who ever vaccinated their pets did so in the past year based on the requirement of the MOH for annual vaccination. Respondents who completed education at college and university levels were less likely to ever vaccinate their pet, χ^2^(2, N = 515) = 9.837, *p <* .*01*). The percentage of respondents that vaccinated pets in the last year did not differ by education, gender, or age group. Fig 3 shows that less than half of the respondents reported they had vaccinated livestock at any time. Fig 4 shows that slightly above one-third of respondents reported they vaccinated livestock in the last year. The percentage of respondents that vaccinated livestock in the last year differ by education. Respondents who completed education at college and university levels were less likely to vaccinate livestock at any time compared to respondents who completed education at lower education levels χ^2^(2, N = 100) = 10.07, *p <* .*01*)). Overall, only 168 (27.2%) respondents reported they had vaccinated animals in the government’s anti-rabies program.

Of 314 respondents who reported vaccinating pets at any time, 261 (83.1%) reported they vaccinated dogs, 28 (8.9%) vaccinated cats, and 6 (1.9%) vaccinated other pets. Of 189 respondents who reported vaccinating livestock at any time, 44 (23.3%) vaccinating sheep or goat, respectively, 9 (4.8%) vaccinated pigs, and 18 (9.5%) vaccinated cattle.

### Knowledge of the Ministry of Health’s anti-rabies programs

Several reasons were given by the respondents (118, 62.2%) for failing to vaccinate livestock; most commonly, respondents did not know about the Government’s anti-rabies vaccination program (48, 40.7%), vaccination took too long (44, 37.3%), transportation problems to bring the animals to the vaccination site (13, 11.0%), and not being at home when the vaccination team was in the area (11, 9.3%). Almost half of the respondents, 281 (45.5%), also stated that the MOH should remain in charge of the anti-rabies program, 84 (13.6%) felt that animal owners should control the program, and 47 (7.6%) respondents felt the Ministry of Agriculture should be in charge. Apart from the vaccination program, generally, less than 20% of respondents knew about the other programs, except for about one third that was also aware of the mongoose trapping and stray dog control programs. Table 4 shows respondents’ awareness of the anti-rabies programs in the MOH.

**Table 4:** Awareness of the Ministry of Health’s anti-rabies programs.

Anti-rabies Program	Number	%
Anti-rabies vaccination	338	54.8
Stray dog control	241	39.1
Mongoose trapping	197	31.9
Public education on rabies	89	14.4
Investigating reports of persons bitten	74	12.0
Medical care, evaluation and rabies protection of persons bitten by animals	57	9.2
Rabies surveillance in people and animals	28	4.5
None of the programs	108	17.5

### Evaluation of the Ministry of Health’s vaccination team

The MOH’s vaccination team was mostly rated as helpful, friendly, informative, and knowledgeable. Table 5 shows the respondents evaluation of the anti-rabies vaccination team in the Ministry of Health.

**Table 5:** Respondents’ evaluation of the vaccination team.

Description	Number	%
Helpful	138	33.3
Friendly	95	22.9
Informative	91	22.0
Knowledgeable	91	22.0
Professional	73	17.6
Unable to provide information/answers	24	5.8
Uncooperative	21	5.1

### Recommendations to improve the vaccination program

Increasing education programs, controlling the mongoose population, and increasing the number of times that the vaccination program is conducted in each year were the most frequent recommendations made to improve the rabies control program. To disseminate information about the anti-rabies program, respondents most commonly suggested using the MOH’s public address system in communities (82, 13.3%) and radio announcements (72, 11.7%).

## Discussion

This is the first study conducted in Grenada to assess KAP regarding rabies and to assess the public’s feedback on the MOH’s anti-rabies programs. While this is the first study to assess the rabies control program in Grenada, the findings from other studies and clinical data indicate that the Grenadian population is at risk for rabies. A study conducted in Grenada from 2011–2013 on rabies prevalence in mongooses that were trapped found that about 0.5–1.5% of the animals were infected with the rabies virus [13]. Another study shows that of 173 animals tested between 2001–2016, 64 (36.4%) tested positive for the rabies virus with the highest prevalence in dogs and mongooses [14]. Both dogs and mongooses are likely to come in contact with humans and, therefore, pose a risk of transmission of the virus to humans through bites from infected animals. In 2015, a total of 384 cases were reported to the MOH of persons bitten by dogs (314 cases reported by community clinics, 70 cases reported by hospitals) [15]. As such, this study provides information that can be used to guide the MOH and its partner institutions in enhancing the efforts to control rabies in Grenada. The data can also be used as a baseline for comparison with future studies to determine whether there were changes in knowledge and behaviors relating to rabies. Other countries in the Caribbean region may refer to these findings to identify at-risk populations and to guide in the development of strategies to break the transmission cycle.

Prior to conducting this study, the proportion of households with animals that are susceptible to rabies–that is, animals that can be infected with the rabies virus—was not established in Grenada. The findings of this study show that about 62% of households have animals that are susceptible to the disease. This percentage can be used as a reference for the scope that should be covered by the MOH’s anti-rabies program and to inform planning and resource allocation. Additionally, this reference can be used to plan sampling for other studies, such as, follow up studies to monitor changes in KAP among animal owners over time or immediately following interventions.

During the time of this study, the government operated laboratory, with capacity to analyze samples from animals, was not functional. However, the services were provided at St. George’s University veterinary laboratory. This restricts capacity to handle post-exposure emergencies, particularly, by animal owners with limited resources to pay for the services at the University. Meanwhile, however, the MOH continues to purchase and administer rabies vaccine to individuals who are believed to be at risk after being bitten by animals. As mentioned above, this measure is not cost-effective. Efforts should be made to repair the laboratory in the shortest time period.

In keeping with the Dog Control and Regulation Act of 2002, registration of dogs and vaccination of domestic animals are the two approaches that were instituted by the MOH to manage disease transmission [14]. A study conducted by Keku et al. (2016) on stray dogs in Grenada found that more stray dogs were being captured and that the rate of dog registration and vaccination had decreased significantly between 2008–2012 [16]. As such, the provisions of the Control and Regulation Act of 2002 should be fully exercised, requiring dogs to be registered [14] and, thus, providing an avenue for better coordination and monitoring of the reach of the vaccination program. Free roaming dogs are at high risk for contracting rabies from wild animals, such as mongoose, and can transmit the disease to humans [16]. Controlling the population of free-roaming dogs is, therefore, a critical step in breaking the transmission line. Registration of other animals, such as livestock, should also be considered for ease of mobilization for vaccination and to monitor coverage.

The findings show that, generally, respondents were not very knowledgeable about animals that are susceptible to rabies. Dog was identified by the majority of respondents, however, only a few respondents identified bat and there was low responses in identifying most of the other animals, such as pig and cattle. Most respondents also had limited knowledge about the signs of rabies in animals. Males were more likely to correctly identify animals that are susceptible to rabies as well as the signs of rabies in animals. This finding may indicate differences in access to information about rabies or the influence of livelihood practices—males are generally more involved in animal husbandry. In any case, the MOH should conduct an evaluation of education programs to ensure that there is equity in opportunities to learn about the disease. Further studies can also be conducted to investigate factors that may influence knowledge about rabies among males and females. Hunters, farmers, forest rangers, and other groups that may readily come in contact with animals are at higher risk and should be targeted for education programs. Increasing the level of knowledge about signs of rabies can also lead to increased reporting to surveillance.

There was also very low participation in the vaccination program, especially by respondents who owned livestock. Apart from the MOH, vaccination is also provided by private veterinarians and at the animal clinic at St. George’s University suggesting options for participating in vaccination programs. Only education was found to be associated with participation in the animal vaccination program. Respondents with higher levels of education–college and above—were also less likely to vaccinate animals as compared to respondents who completed education at lower institutions. This information is critical for the MOH to guide in targeting strategies for rabies education programs.

Apart from the vaccination program, there was limited awareness about the other anti-rabies programs in the MOH. The results show that there was little awareness about the other anti-rabies programs conducted by the MOH, including stray dog control, mongoose trapping, public education, investigating reports of persons bitten by animals, and rabies surveillance. This finding is also interesting, given that the majority of respondents also stated that they would call the MOH if they were bitten by an animal or suspected that an animal had rabies. The findings may indicate that, despite calling the MOH, the public was still uncertain about the courses of action that may be taken. In addition, there may be a lack of awareness by some community Health professionals of the existing rabies treatment protocol. The MOH should incorporate information about all the anti-rabies programs in a comprehensive education campaign. This step can also contribute to increase confidence in the MOH’s strategies to address rabies in various ways.

While vaccination is encouraged to protect the health of the public, there is no legal mandate for public compliance. As such, to address the low participation in animal vaccination programs, the MOH would need to develop and utilize strategies that are appealing to the public. The current rate of participation in vaccination is not sufficient to achieve herd immunity. The WHO cited that 70% of dogs in an area must be vaccinated to achieve heard immunity [17]. The MOH may consider establishing a committee to develop a strategic plan and oversee interventions to address gaps in knowledge and practices.

Among the main reasons suggested for the low vaccination coverage were unawareness of the anti-rabies program and the long time taken to vaccinate animals. Challenge in transporting animals to vaccination sites was also mentioned. As such, the MOH may need to consider the practicality and feasibility of providing vaccination services on farms and other convenient locations. Consideration should also be given to extending the vaccination programs to weekends and evenings to accommodate employees that work during regular hours. A fee-for-service system may be considered to support the anti-rabies program.

The MOH can also explore the possibility of utilizing Oral Rabies Vaccine (ORV). ORV was used in the United States from the mid-1990s to date to prevent and control wildlife rabies [18]. Investigations may be conducted to determine how factors such as climatic conditions, animal species, territorial behavior patterns and physiological characteristics of animals may affect the suitability of this initiative for the Grenada setting.

In many ways, the findings in this study were similar to results in other countries [5, 7, 14]. In studies conducted in 2013 and 2014–2015 on KAP related to rabies in Ethiopia, it was found that while most respondents were aware of rabies, the majority did not have accurate knowledge about how rabies was transmitted, signs of the disease and prevention and treatment measures [19,20]. Dogs and cats were most commonly identified as animals involved in transmission of the virus and the majority of respondents also correctly stated that the disease can be transmitted through bites from animals [19,20]. These findings in Ethiopia reflect similar knowledge of respondents in this study. In another study conducted in Tanzania in 2009–2010 among 5,141 respondents, a similar result to Grenada was also achieved with regard to the low proportion of respondents who vaccinated animals [9]. About half of the respondents, 51.0%, had vaccinated dogs and several gaps were found in knowledge and practices that were associated with socio-economic status [9].

While most of the respondents in this study indicated that their primary place of learning about rabies was school/work, the findings also show inconsistencies in their knowledge that may reflect on the content of the education programs. Some insights were provided in a study that was conducted in Bangladesh in 2014 with teenage students in two high schools [8]. The researchers found that there was a low level of knowledge about rabies and poor handling of pets by the students and that increased the risk of transmission of the disease [8]. Following a deliberate and well-planned education intervention, the level of knowledge and practices improved. The MOH can benefit from the Bangladesh experience, noting that similar result can be achieved from implementing a planned program in schools in Grenada.

There were limitations in conducting this study in Grenada. The representation of male and females in the study were consistent with the census (2011) proportions, however, the overwhelming majority of respondents had completed education at primary and secondary school levels. This inconsistency may be as a result of the time of day that the survey was conducted. People with lower education may likely be unemployed and at home during regular working hours. For studies in the future, one approach that can be taken to reduce this issue is to designate specific hours for data collection.

Some questions need to be revised to be more specific and to improve the quality of the data. For example, the option of “school/work” should be separated to give a clearer indication of the specific place where the respondent first learned about rabies. Prior to use in another study, the questionnaire should be reviewed and compared with the tools used in other countries. The questionnaire can then be refined and used as a standard tool across the region allowing for comparability and collation of findings.
